# 高频胸壁振荡排痰仪对单孔胸腔镜肺叶切除术后肺功能的影响

**DOI:** 10.3779/j.issn.1009-3419.2018.12.05

**Published:** 2018-12-20

**Authors:** 雪娟 朱, 元骏 程, 文涛 杨, 勇兵 陈, 立 施

**Affiliations:** 215004 苏州，苏州大学附属第二医院胸心外科 Department of Cardiothoracic Surgery, the Second Hospital Affiliated to Soochow University, Suzhou 215004, China

**Keywords:** 单孔, 胸腔镜, 高频胸壁振荡排痰仪, 肺功能, Single port, Video-assisted thoracoscopic surgery, High-frequency chest wall oscillatory, Lung function

## Abstract

**背景与目的:**

高频胸壁振荡排痰仪（high-frequency chest wall oscillatory, HFCWO）是一种新型的辅助排痰装置，但其相关的作用效果仍不明确。本研究通过比较高频胸壁振荡排痰仪使用前后患者排痰量的变化，进一步探讨高频胸壁振荡排痰仪对单孔胸腔镜（single port video-assisted thoracoscopic surgery lobectomy, S-VATS）肺叶切除术后肺功能和血气分析的影响。

**方法:**

收集2017年1月-2017年12月于苏州大学附属第二医院行S-VATS肺叶切除术患者共90例，随机分为实验组和对照组，每组45例。实验组患者采用高频胸壁振动排痰仪排痰，对照组采用常规拍背排痰。检测两组术后前5天的排痰量以及两组术前、术后第7天肺功能和动脉血气分析。

**结果:**

实验组治疗后排痰量均多于对照组，且术后第1天、第2天、第3天比较差异有统计学意义（*P* < 0.05）。两组术前第1秒用力呼气容积（forced expiratory volume in one second, FEV_1_）、用力肺活量（forced vital capacity, FVC）及PaO_2_比较差异无统计学意义（*P* > 0.05）；术后两组FEV_1_、FVC及PaO_2_均较术前下降（*P* < 0.05）；但实验组FEV_1_、FVC及PaO_2_均高于对照组（*P* < 0.05）；两组术前、术后PaCO_2_比较差异无统计学意义（*P* > 0.05）。

**结论:**

S-VATS肺叶切除术后给予高频胸壁振荡排痰仪可以显著提高排痰量，改善肺功能和缺氧状态，临床疗效满意。

## Abstract (en)

**Background and objective:**

It has been confirmed that high-frequency chest wall oscillatory (HFCWO) is a new type of auxiliary sputum discharge device. However, up to now, the specific therapeutic effect of HFCWO is still uncertain. This study aimed to compare the changes of the sputum volume before and after the treatment of HFCWO, and to investigate the effect of HFCWO on lung function and arterial blood gas analysis after single port video-assisted thoracoscopic surgery lobectomy (S-VATS).

**Methods:**

A total of 90 patients with S-VATS lobectomy were collected in the Second Affiliated Hospital of Soochow University from January 2017 to December 2017, which were randomly divided into the experimental group with HFCWO (*n*=45) and the control group (*n*=45) with routine clapping, respectively. The sputum volume of the two groups was measured 5 days before operation. Lung function and arterial blood gas analysis was measured before and 7^th^ days after surgery.

**Results:**

The sputum volume was higher in the experimental group than that of the control group after surgery, there was statistically significant difference for the first three days (*P* < 0.05). There was no statistically significant difference between the two groups in forced expiratory volume in one second (FEV_1_), forced vital capacity (FVC) and oxygen partial pressure (PaO_2_) before surgery (*P* > 0.05); Compared with those before surgery, FEV_1_, FVC and PaO_2_ decreased in both groups after surgery (*P* < 0.05); However, FEV_1_, FVC and PaO_2_ in the experimental group were higher than those in the control group (*P* < 0.05); There was no statistically significant difference in preoperative and postoperative partial pressure of carbon dioxide (PaCO_2_) between the two groups (*P* > 0.05).

**Conclusion:**

HFCWO can significantly increase the amount of sputum excretion, improve lung function and alleviate hypoxia status after S-VATS lobectomy. This study provides a promising approach for HFCWO toward hypoxia status after S-VATS lobectomy.

近年来术后肺功能障碍仍然是心肺手术潜在危险并发症之一^[1]^。手术中麻醉、肺手术创伤等使患者气道分泌物增加，排痰效果不佳时，将加重患者病情，严重时甚至会导致低氧血症的发生，威胁患者生命安全^[2]^。提高排痰效果、促进气道通畅是改善患者术后肺功能的关键。单孔胸腔镜微创肺癌根治术具有切口美观、恢复快、创伤小以及对肺功能影响小的优点^[3]^。高频胸壁振动排痰仪（high-frequency chest wall oscillatory, HFCWO）通过压缩和放松胸壁产生振荡体积模拟咳嗽，有效地清除气道分泌物改善通气和氧合^[4]^。本研究给予单孔胸腔镜（single port video-assisted thoracoscopic surgery lobectomy, S-VATS）肺叶切除术患者外用HFCWO治疗，探讨其对减轻术后肺功能障碍的作用。现报告如下。

## 1 资料与方法

### 1.1 临床资料

选取2017年1月-2017年12月苏州大学附属第二医院行S-VATS肺叶切除术的肺癌患者90例，随机分为实验组和对照组，每组45例。实验组患者术后采用HFCWO排痰，对照组采用常规拍背排痰。实验组患者中位年龄42岁，男性23例，女性22例。对照组中位年龄39岁，男性22例，女性23例。两组性别、年龄、手术类型等方面比较差异无统计学意义（*P* > 0.05），具有可比性。本研究经医院伦理委员会批准，且患者及家属签署知情同意书。纳入标准：①年龄 < 70岁，无呼吸系统原发疾病；②肺部无创伤史，肺功能正常，无心脑血管疾病等；③非小细胞肺癌，术前未行放疗。排除标准：①年龄 > 70岁；有肺部基础疾病；②胸部创伤病史；③1个月内发生过心肌梗死者；④凝血功能障碍、肝肾功能不全。

### 1.2 手术及方法

#### 1.2.1 手术过程

两组术前准备和手术体位均相同，均采取双腔气管插管，健侧单肺通气，患者侧卧位。手术方式均为S-VATS，常规行肺叶切除及纵隔肺门淋巴结清扫。腋前线第4或5肋间，切口长度3 cm-4 cm，器械、镜头均经此孔进行操作。术中尽量避免对正常肺组织的牵拉和挤压。

#### 1.2.2 使用方法

所有患者术后第1天开始均进行排痰治疗，并常规抗感染治疗、雾化吸入治疗、平喘、止痛药物治疗等。对照组采用常规拍背排痰，2次/d，上午、下午各1次，5 min/次。实验组采用HFCWO排痰，使用中国常州思雅医疗器械公司生产的YSQ01C型HFCWO治疗，患者治疗前先穿上可充气夹克背心，并根据患者实际体型为其调整背心松紧度，可放入一掌最佳。将患者夹克背心与脉冲气体发生器使用管路连接，治疗模式为成人模式（频率9 Hz-11 Hz-13 Hz-10 Hz），治疗时间设置为10 min。患者治疗时直接按下加压键，夹克背心将有规律的进行充气、产生振动，辅助排痰治疗。

### 1.3 检测指标

术前及术后第7天分别行肺功能检测，包括第1秒用力呼气容积（forced expiratory volume in one second, FEV_1_）和用力肺活量（forced vital capacity, FVC）；血气分析，包括二氧化碳分压（partial pressure of carbon dioxide, PaCO_2_）和氧分压（oxygen partial pressure, PaO_2_）。术后排痰量以及肺部并发症等。

### 1.4 统计学方法

采用SPSS 17.0软件包进行统计学分析，计数资料采用率（%）表示，组间比较采用*χ*^2^检验；计量资料采用均数±标准差（Mean±SD）表示，组间比较采用*t*检验，以*P* < 0.05为差异有统计学意义。

## 2 结果

### 2.1 两组术中风险因素比较

比较两组患者手术过程中可能影响术后肺功能的风险因素，包括手术时间、麻醉时间、术中出血量，两组比较差异均无统计学意义（*P* > 0.05）。见表 1。

**1:** 两组患者术中情况比较（Mean±SD） Comparison of the intraoperation (Mean±SD)

Group	*n*	Operation time (min)	Anesthesia time (min)	Blood loss (mL)
Experimental group	45	62.333±8.761	106.875±8.425	48.571±6.268
Control group	45	70.222±7.102	106.750±2.314	55.000±10.000
*t*		-2.098	-0.024	-1.441
*P*		0.052	0.981	0.175

### 2.2 两组术后排痰量比较

实验组治疗后痰量均高于对照组，第1、2、3天差异均有统计学意义（*P* < 0.05）。见表 2。

**2:** 两组患者术后排痰量比较（Mean±SD, mL） Comparison of the postoperative sputum volume (Mean±SD, mL)

Group	*n*	Day 1	Day 2	Day 3	Day 4	Day 5
Experimental group	45	14.000±2.767	15.000±1.633	17.500±1.871	16.000±2.380	5.867±2.116
Control group	45	5.667±1.633	10.714±2.430	14.167±1.472	15.871±1.345	6.286±2.215
*t*		6.371	3.873	3.340	0.138	-0.370
*P*		0.000	0.002	0.006	0.892	0.718

### 2.3 两组肺功能情况比较

两组术前FEV_1_和FVC比较差异无统计学意义（*P* > 0.05）；术后第7天两组FEV_1_和FVC均较术前下降，但实验组高于对照组（*P* < 0.05）。见表 3。

**3:** 两组手术前后肺功能情况比较（Mean±SD, %） Comparison of the pulmonary function in pre-operation and post-operation (Mean±SD, %)

Group	*n*	Pre-operation			7^th^ day post-operation	
		FEV_1_	FVC		FEV_1_	FVC
Experimental group	45	72.857±7.034	76.567±5.396		66.700±1.287	62.629±1.098
Control group	45	71.900±6.155	76.214±3.050		63.042±1.620	60.014±1.635
*t*		0.271	0.151		4.677	3.511
*P*		0.791	0.883		0.001	0.004
FEV_1_: Forced expiratory volume in one second; FVC: Forced vital capacity.						

### 2.4 两组血气分析指标比较

两组术前PaO_2_比较差异无统计学意义（*P* < 0.05）；两组术后PaO_2_均低于术前，但实验组高于对照组（*P* < 0.05）。两组手术前、术后PaCO_2_比较差异无统计学意义（*P* > 0.05）。见表 4。

**4:** 两组手术前后血气分析指标比较（Mean±SD, mmHg） Comparison of the blood gas analysis in pre-operation and post-operation (Mean±SD, mmHg)

Group	*n*	Pre-operation			7^th^ day post-operation	
		PaO_2_	PaCO_2_		PaO_2_	PaCO_2_
Experimental group	45	82.114±3.647	43.843±1.633		72.400±2.080	42.086±1.306
Control group	45	79.143±1.364	40.700±2.096		67.043±3.413	41.257±2.342
*t*		2.019	2.119		3.546	0.878
*P*		0.066	0.056		0.040	0.434

### 2.5 两组术后并发症及住院时间比较

两组术后并发症及住院时间比较差异均无统计学意义（*P* > 0.05）。见表 5。

**5:** 两组术后并发症及住院时间比较 Comparison of the complications and hospital stay in post-operation

Group	*n*	Complication				LOS (Mean±SD, d)
		Pneumonia	Massive hemorrhage	Malignant arrhythmia	RDS	
Experimental group	45	1	1	2	3	6.600±1.195
Control group	45	2	1	1	2	6.889±1.265
*χ*^2^/t		0.794				-1.114
*P*		0.851				0.268
RDS: respiratory distress syndrome; LOS: length of stay.						

## 3 讨论

胸腔镜下肺叶切除术在肺癌治疗中的应用越来越广泛。与开胸手术相比，胸腔镜微创手术具有术后疼痛轻、并发症少、恢复快的优点^[5]^。随着技术水平成熟和相关器械的研发以及术前3D影像学技术的发展，处理肺门血管更加方便。S-VATS肺叶切除术治疗肺癌有日益替代双孔或多孔VATS的趋势^[6, 7]^。S-VATS最大的特点在于仅有一个3 cm-4 cm手术切口，胸壁的损伤小，其同时具备观察孔及操作孔的功能，不影响实际的操作。

患者因高龄、合并COPD等基础疾病、术前呼吸道准备不足；术中手术时间过长、健侧肺过度通气、气管插管和机械通气、过早拔除气管插管均可降低或影响排痰功能。肺内大量分泌物会影响支气管纤毛柱状上皮的功能、血氧饱和度、通气血流比值等。术后患者因切口疼痛不愿意主动咳嗽导致咳痰障碍。故及时促进患者排痰、改善患者气道状况是术后康复的重点。为减少高龄对本研究结果的影响，本次入组的患者均为体检发现，具有住院年龄小、手术耐受性强的特点。

人工叩背法是以往辅助排痰的常用方式，其效果往往不佳。HFCWO通过一定的规律充气和放气，挤压和放松胸壁，诱发吸气气流，改变粘液表面剪切力，同时刺激纤毛，提高纤毛摆动频率，从而使呼吸道分泌物得到松解，粘液的物理性状发生改变，从而有利于分泌物排出^[8, 9]^。本研究中实验组治疗后1 d、2 d、3 d排痰量均高于对照组，差异有统计学意义（*P* < 0.05），说明采取HFCWO辅助排痰效果更佳。使用HFCWO辅助排痰时，机器自动充气夹克背心辅助排痰，既可有效减少医院人力资源消耗，又能促进振荡的均匀性，提升排痰效果。

肺功能的影响因素很多，如吸烟、高龄、性别、身高、严重胸廓畸形、基础心肺疾病等。术后早期患者往往因为疼痛而限制胸廓的运动，且肺癌患者肺功能变化与术后并发症和生存率关系密切^[10]^。因为术后7 d以内也是呼吸相关并发症发生率较高的时期，因此测定术后第7 d患者肺功能的情况具有重要意义。FEV_1_指最大吸气至肺总量后第1秒内用力的呼气量，既是容量测定，也是1 s内的平均流速测定，是肺功能受损的主要指标；FVC是指尽力最大吸气后，尽力尽快呼气所能呼出的最大气量，可以反映较大气道的呼气期阻力。因此FEV_1_和FVC可作为反映肺通气功能程度的良好指标^[11]^。本研究结果显示，两组术前FEV_1_和FVC比较差异无统计学意义；术后第7天两组FEV_1_和FVC均较术前下降，但实验组FEV_1_、FVC均高于对照组，差异有统计学意义（*P* < 0.05）。提示HFCWO显著改善了术后肺通气功能。

动脉血气分析主要用于对患者缺氧状况、二氧化碳潴留和酸碱代谢状态的判断，评估患者缺氧情况和肺泡通气情况，从而反映患者肺功能水平^[12-14]^。本研究结果显示，术前两组患者PaO_2_比较差异无统计学意义，术后两组患者PaO_2_较术前下降。提示缺氧状况在术后有所加重，但与对照组相比，实验组能够显著改善患者缺氧状态，差异有统计学意义（*P* < 0.05）。本研究发现，两组术前及术后PaCO_2_均无统计学差异（*P* > 0.05）。这是因为CO_2_的扩散系数约为O_2_的20倍，通常PaO_2_先于PaCO_2_发生变化，或出现阻塞性通气障碍时PaCO_2_才发生异常。

综上所述，在S-VATS肺叶切除术后给予HFCWO治疗可以提高排痰量，显著改善患者肺功能和缺氧状态，且不会增加肺部感染、无胸腔大量出血、恶性心律失常、呼吸窘迫综合征等并发症的发生率，不影响住院时间，具有较好的安全性。
